# Ado-tratuzumab emtansine beyond breast cancer: therapeutic role of targeting other HER2-positive cancers

**DOI:** 10.3389/fmolb.2023.1165781

**Published:** 2023-05-11

**Authors:** Yang Zheng, Jiayu Zou, Chen Sun, Fu Peng, Cheng Peng

**Affiliations:** ^1^ State Key Laboratory of Southwestern Chinese Medicine Resources, Chengdu University of Traditional Chinese Medicine, Chengdu, Sichuan, China; ^2^ Chengdu University of Traditional Chinese Medicine, Chengdu, Sichuan, China; ^3^ Sichuan University, Chengdu, Sichuan, China

**Keywords:** T-DM1, HER2^+^, cancer, therapeutic potential, targeted therapy

## Abstract

Ado-trastuzumab emtansine (T-DM1) is an antibody–drug conjugate approved by the FDA in 2013 for advanced HER2-positive breast cancer treatment exhibiting promising clinical benefits. However, HER2 overexpression and gene amplification have also been reported in other cancers like gastric cancer, non-small cell lung cancer (NSCLC), and colorectal cancer*.* Numerous preclinical studies have also revealed the significant antitumor effect of T-DM1 on HER2-positive tumors. With the advancement in research, several clinical trials have been conducted to investigate the antitumor effect of T-DM1. In this review, we briefly introduced the pharmacological effects of T-DM1. We reviewed its preclinical and clinical studies, especially on other HER2-positive cancers, establishing what has been encountered between its preclinical and clinical studies. In clinical studies, we found that T-DM1 has a therapeutic value on other cancers. An insignificant effect was observed on gastric cancer and NSCLC, inconsistent with the preclinical studies.

## 1 Introduction

Breast cancer (BC) is the leading cause of cancer incidence worldwide, affecting nearly 2.3 million people and causing nearly 680,000 deaths each year. Although the treatment of BC has improved after the advent of targeted therapy, it remains a serious disease worldwide (Sung et al., 2021). In clinical studies, the early treatment of BC was mainly through cytotoxic chemotherapy (Tinoco et al., 2013), which has many limitations (Chiuten et al., 1983). According to the immunophenotypic classification, BC is categorized into hormone receptor-positive (including estrogen receptor and progesterone receptors), HER2 overexpressed BC, and triple-negative breast cancer (TNBC) (Hammond et al., 2010; Garrido-Castro et al., 2019). HER2^+^ BC is one of the more aggressive BC phenotypes among all BC phenotypes. HER2 is an ErbBs or type I receptor tyrosine kinase family member, also known as ErbB-2 and Neu, structurally related to the epidermal growth factor receptor (EGFR) (Elizalde et al., 2016). The HER2 gene encodes a 185-kDa heavy transmembrane glycoprotein, composed of an extracellular ligand-binding domain, a transmembrane domain, and an intracellular receptor domain (Schlessinger, 2002). In 1983, Slamon et al. (1987) first found that the HER2 gene was amplified in human BC cell lines; this aspect was studied over the past 4 decades. Several studies have shown that HER2 regulates cell proliferation, tumor growth, cell migration, invasion, and angiogenesis through phosphatidylinositol 3-kinase (PI3K) and mitogen-activated protein kinase pathways (Atalay et al., 2003; Riecke and Witzel, 2020). Immunohistochemical analysis exhibited that overexpression of HER2 in BC cells could increase the number of HER2 receptors in each cell to nearly 2 million (Derakhshani et al., 2020). Targeted therapy with HER2, especially with the HER2 antibody trastuzumab, has become the standard treatment for HER2^+^BC. Trastuzumab, a drug with successful implications in targeted therapy, shows an antitumor effect on breast cancer with HER2 overexpression, while having no significant effect on tumors with normal HER2 expression (Cameron et al., 2017; Tolaney et al., 2019; Derakhshani et al., 2020). However, clinical studies revealed serious acquired resistance to trastuzumab in its clinical use (Vivekanandhan and Knutson, 2022), restricting the clinical use of trastuzumab, and a new drug is urgently required to solve this problem.

With the unremitting efforts of researchers, an antibody–drug conjugate (ADC) named ado-trastuzumab emtansine (T-DM1 or Kadcyla), with a powerful antitumor effect, has been developed. In 2020, Montemurro et al. (2020) conducted the KAMILLA study involving 398 patients with HER2^+^ advanced or brain–metastatic breast cancer and yielded promising results, pointing toward the potential of T-DM1 in treating HER2^+^ metastatic BC. Conferring to the results of clinical studies, T-DM1 shows a strong antitumor effect, and it can obtain encouraging results in treating BC and mBC (Clark et al., 2021; Daniels et al., 2021). Interestingly, HER2 overexpression is not limited to BC. Genome sequencing has revealed the presence of HER2 amplification in other types of solid tumors, which has inspired more research in this direction. More recently, studies have shown the therapeutic effect of T-DM1 on not only HER2^*+*^ BC but also on other HER2^*+*^ cancers (Table 1) (Peters et al., 2019; Rinnerthaler et al., 2019).

**TABLE 1:** Clinical trials of T-DM1 in various cancers.

Trial	Phase	HER2 criteria	Efficacy	Adverse event	Cancer type	References
NCT01641939	II/III	IHC2^+^ and an ISH-positive or IHC3^+^, regardless of ISH status	OS: 7·9 vs. 8.6 months (HR 1·15, 95% CI 0 · 87–1·51, and *p* = 0·86) PFS: 2.7 vs. 2.9 months (HR, 1·13, 0·89–1·43, and *p* = 0·31)	Any grade 97% vs. 97%, grade ≥3 60% vs. 70%	Gastric cancer	Thuss-Patience et al. (2017)
NCT02675829	II	Exon 20 insYVMA, insGSP, or insTGT; single-base pair substitutions L755A, L755S, V777L, V659E, or S310F; or other likely activating mutations	ORR: 44% (95% CI, 22%–69%) PFS: 5 months (95% CI, 3–9 months)	Grade 3:6% and Grade 4:0%	Non-small cell lung cancer	Li et al. (2018)
NCT02289833	II	IHC2^+^ or 3^+^ EGFR mutated or ALK gene rearranged	IHC3^+^ cohort: ORR:20% (95% CI 5.7%–43.7%) IHC2^+^ and 3^+^ cohort: PFS: 2.6 vs 2.7 month (95% CI 1.4–2.8) OS: 12.2 vs 15.3 months (95% CI 3.8–23.3)	Any grades: 92% Grade 3:20% and Grade 4:2%	Non-small cell lung cancer	Peters et al. (2019)
JapicCTI-194620	II	HER2 exon-20 insertion mutations	ORR: 38.1% (90% CI, 23.0%–55.9%, and *p* = 0.2091) PFS: 2.8 months (95% CI, 1.4–4.4 months) OS: 8.1 months (95% CI, 3.5–13.2 months)	Toxicity was mild, with the frequency of adverse events of grade ≥3 being low	Non-small cell lung cancer	Iwama et al. (2022)
NCT03225937	II	IHC3^+^ or IHC2^+^ and an ISH-positive mCRC with RAS (KRAS exons 2, 3, and 4; NRAS exons 2, 3, and 4) wild-type status	ORR: 9.7% (95% CI, 0–28) PFS: 4.1 months (95% CI, 3.6–5.9)	Grade ≥3:1%	Colorectal cancer	Sartore-Bianchi et al. (2020)

To illustrate and provide practical evidence of the antitumor effects of T-DM1 in other cancers, we collected, summarized, and reviewed the most relevant literature in the past 5 years, aiming for the significant therapeutic potential of T-DM1 on all types of cancer treatment in PubMed, Web of Science, *etc* (Dhillon, 2022). Duplicate articles and articles with similar results were excluded. Finally, 96 articles were included after reading the titles, abstracts, and complete manuscripts.

## 2 T-DM1 is an antibody drug approved by the FDA for breast cancer treatment types

### 2.1 The clinical indication of T-DM1

T-DM1 is a novel ADC that was first approved by the Food and Drug Administration (FDA) and European Medicines Agency (EMA) in 2013 for metastatic breast cancer (mBC) treatment (Amiri-Kordestani et al., 2014). In phase III, the EMILIA study involved patients with HER2^*+*^ mBC previously treated with trastuzumab and taxane, and T-DM1 significantly improved progression-free survival (PFS) and overall survival (OS) compared with combination therapy consisting of capecitabine and lapatinib (Verma et al., 2012). In an interim analysis of the KATHERINE trial involving patients with HER2-positive early breast cancer (EBC) and residual invasive disease in the breast or axilla after neoadjuvant therapy with trastuzumab and taxane, T-DM1 provided better prognosis than trastuzumab in these patients (von Minckwitz et al., 2019). Based on this result, the FDA approved T-DM1 as adjuvant therapy for patients with HER2-positive EBC (Hunter et al., 2020).

### 2.2 The mechanism of T-DM1

T-DM1 comprises three components: trastuzumab, linker, and cytotoxic payload—small-molecule microtubule inhibitor DM1 (Xu et al., 2019). As mentioned previously, trastuzumab has a satisfactory targeting effect on the HER2 receptor, but its killing effect on tumor cells is not strong enough due to the acquired drug resistance to trastuzumab. DM1, a small-molecule microtubule inhibitor, is a derivative of maytansine. Maytansine has been found to have a strong killing effect on tumor cells (Lambert and Chari, 2014). However, maytansine can cause a series of side effects in normal tissue cells due to its low selectivity (Xu et al., 2019). Scientists obtain the essence and discard the dregs, adding a sulfhydryl functional group to maytansine (for easier formation of antibody–drug conjugates) and coupling trastuzumab to DM1 with a stable linker-heterobifunctional agent 4-(N-maleimidomethyl) cyclohexane-1-carboxylate (SMCC), which can release effective loads specifically on specific target cells (Su et al., 2021). It was shown that even trastuzumab had undergone MCC derivations and retained its original mechanism of action against the HER2 receptor (Junttila et al., 2011).

**FIGURE 1:**
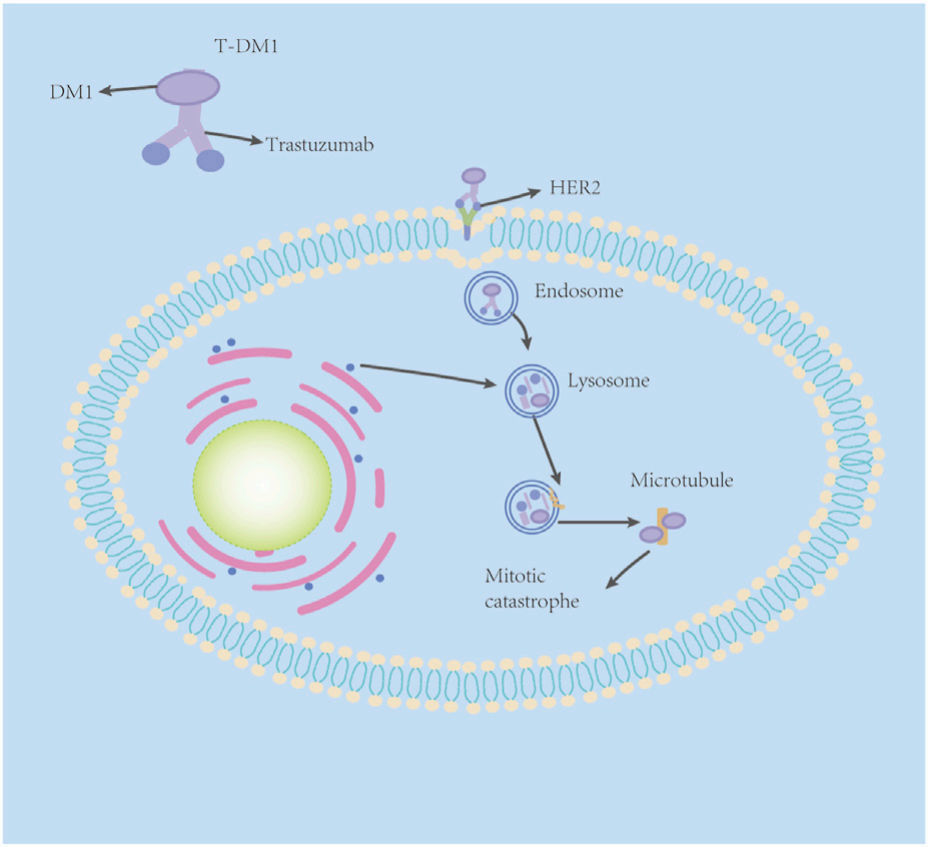
Mechanisms of the action of T-DM1.

The antitumor mechanism of T-DM1 is two-fold. First, it retains the antitumor mechanisms of the humanized monoclonal antibody trastuzumab: HER2 signaling inhibition, inhibition of HER2 extracellular domain shedding, and mediation of antibody-dependent cell-mediated cytotoxicity (ADCC). In addition, through DM1-mediated cytotoxicity, T-DM1 partially binds to HER2*,* specifically through trastuzumab to form the HER2–T-DM1 complex (Barok et al., 2014), which is then internalized into early endosomes via receptor-mediated endocytosis. A fraction of the internalized vesicles mature to deliver the T-DM1 receptor complex to cell lysosomes, and the HER2–T-DM1 complex in the other fraction of vesicles is recycled back to the plasma membrane (Figure 1) (Hunter et al., 2020). In the lysosome, trastuzumab is proteolytically degraded to liberate the lysine–MCC–DM1 moiety (Barok et al., 2014; Hunter et al., 2020). Next, the lysine–MCC–DM1 moiety will enter the cytoplasm to exert cytotoxic effects, but because the lysine–MCC–DM1 moiety is structurally a lysine derivative with a positive charge at physiological pH, it cannot pass directly over the lysosomal membrane structure by osmosis (Erickson et al., 2006). The lysine–MCC–DM1 moiety must be transported into the cytoplasm by various lysosomal membrane transporters to exert cytotoxic effects (Hamblett et al., 2015). After entering the cytoplasm, the lysine–MCC–DM1 moiety plays a similar role to that of vinca alkaloid vincristine, which can effectively bind to tubulin, and then prevent the polymerization of tubulin, leading to failure of spindle assembly, cell cycle arrest in the metaphase, multinucleated cells and abnormal mitoses, and finally leading to mitotic catastrophe (Issell and Crooke, 1978).

## 3 Oncogenic activation of HER2

A low expression of HER2 in normal tissue cells of the human body is usually inevitable for the development of the liver, kidney, breast, lung, ovary, central nervous system, and other tissues (Maximiano et al., 2016). There are three main mechanisms of oncogenic activation of HER2: first, overexpression and amplification of HER2; second, HER2 receptor mutation activation; third, inhibition of phosphatase activity (Herter-Sprie et al., 2013). Among them, HER2 amplification and overexpression are the most common oncogenic activation mechanisms of HER2 in cancer (Mishra et al., 2017). Due to the good targeting effect on HER2 and cytotoxicity, T-DM1 has made remarkable achievements in treating HER2-positive BC.

## 4 T-DM1 plays a promising therapeutic role on other types of cancer

### 4.1 Gastric cancer

As one of the most malignant tumors among all cancer types, gastric cancer (GC) ranks fifth in incidence and fourth in case fatality; more than 1 million new cases of GC were reported in 2020. It was estimated that about 769,000 people died from GC, which means that every 13 deaths worldwide contained one GC case in 2020 (Sung et al., 2021). Studies have shown that HER2 overexpression or amplification was observed in 20% of GC patients, and the HER2-positive rate of gastroesophageal junction (GEJ) cancer (32.2%) was higher than that of gastric tumors (21.4%) (Van Cutsem et al., 2015).

Therefore, early studies have been conducted to determine the effects of T-DM1 in GC. In 2011, Barok et al. (2011) obtained encouraging results using four human GC cell lines: OE-19, MKN-7, N-87, and SNU-216. They confirmed HER2 gene amplification in all four cell lines through fluorescence *in situ* hybridization (FISH) analysis. Moreover, the flow cytometry assay exhibited the overexpression of HER2 protein in N-87 and OE-19, similar to the BC cell line SKBR-3. However, about 1/3 was detected in MKN-7 and SNU-216 cells. In addition, the results first elucidated the therapeutic potential of T-DM1 on HER2*+* GC *in vitro* and indicated that T-DM1 had a better antiHER2*+* GC bioactivity than trastuzumab. Following it, Yamashita-Kashima et al. (2013) investigated whether a combination of pertuzumab and T-DM1 would enhance the antitumor effect of T-DM1 *in vivo*, by utilizing five human GC cell lines: NCI-N87, SNU-16, SCH, MKN-28, and 4-1ST to construct GC xenograft models. They found overexpression of the HER2 gene in NCI-N87, 4-1ST, and SCH cell lines (IHC3+ or 2+, FISH+: HER2/CEP17 ratio 2.0) and low expression in SNU-16 and MKN-28 cell lines (IHC 1+ or 0, FISH+: HER2/CEP17 ratio 2.0). The results indicated that 20 mg/kg of T-DM1 exhibited significant antitumor activity, with tumor volume reduction observed in the HER2-upregulation group, but not in the HER2-downregulation group. Thus, they used a positive control with T-DM1 to evaluate the efficacy of the combination therapy of T-DM1 and pertuzumab. The combination of T-DM1 and pertuzumab showed notably enhanced antitumor activity (*p* < 0.05) by enhancing the signaling inhibition of the *HER* family. Furthermore, the antitumor efficacy of T-DM1 against NCI-N87 was much stronger than that of trastuzumab used at the maximum effective dose in previous studies (Fujimoto-Ouchi et al., 2007). In SCH cells, trastuzumab did not produce a significant antitumor effect; conversely, T-DM1 could significantly inhibit tumor cell growth. In addition, the combination also potentiated the reduction in the normalized cell index induced by T-DM1 or pertuzumab, enhancing ADCC activity *in vitro*.

However, when researchers supposed that T-DM1 could be used as a second-line drug for the clinical treatment of HER2*+* GC, resistance to T-DM1 was observed in GC. Thuss-Patience et al. (2017) conducted a clinical trial called GATSBY. GATSBY was a three-arm, randomized, open-label, adaptive, and phase 2/3 global study. GATSBY was designed to determine the difference in safety and efficacy between taxane and T-DM1 in previously treated patients with HER2-positive advanced GC. The GATSBY trial was carefully designed, consisting of two parts: the purpose of the phase 2 study was to establish the most suitable T-DM1 dosing regimen from the two T-DM1 dosing regimens, laying the foundation for the phase 3 trial. The phase 3 study was designed to investigate the efficacy and safety of this dose of T-DM1 as compared to that of taxane. In STAGE 1, in the phase 2 study, patients were randomly assigned to the following treatment groups (2:2:1), low-dose T-DM1 group: 3.6 mg/kg every 3 weeks T-DM1; high-dose T-DM1 group: 2.4 mg/kg weekly; T-DM1 taxane monotherapy group: the physician’s choice of taxane (intravenous docetaxel [75 mg/m^2^ every 3 weeks] or intravenous paclitaxel [80 mg/m^2^ every week]) and two administration doses of T-DM1 (2.4 mg/kg weekly compared with 3.6 mg/kg every 3 weeks) were investigated in this phase. In the phase 3 study, patients were randomly assigned to treatment groups (2:1) to receive either T-DM1 (2.4 mg/kg weekly) or the physician’s choice of taxane treatment. The phase 3 study was designed to compare the efficacy and safety of the selected doses of T-DM1 with those of taxanes. The primary endpoint was overall survival (OS). The secondary endpoint was median progression-free survival (PFS). At the end of the experiment, T-DM1 did not show OS superiority. Median OS was 7.9 months (95% CI 6.7–9.5) in the 2.4 mg/kg weekly T-DM1 group and 8.6 months (7.1–11.2) in the taxane group (HR 1.15, 95% CI 0.87–1.51, *p* = 0.86). Median PFS: 2.7 (in the T-DM1 group) (95% CI 1.6–2.7) vs. 2.9 months (in the taxane group) (HR, 1.13, 0.89–1.43, and *p* = 0.31); no significant difference in the objective response rate (ORR) was observed between the two groups. Unfortunately, T-DM1 did not produce the expected effects, despite having a safety profile consistent with its use in metastatic BC; furthermore, there are no data showing the benefit of T-DM1 in patients with HER2-positive advanced GC.

Peter C Thuss-Patience et al. provided some explanations for this result: First, for some reasons, such as discordant HER2 expression between the primary tumor and the metastatic tumor (Peng et al., 2015) or varying HER2 expression after first-line chemotherapy with or without trastuzumab in metastatic disease (Ishimine et al., 2015), the molecular profile of the tumor was altered. Furthermore, it could then lead to alterations and loss of HER2 during progression. Second, GC is a heterogeneous disease. Different from BC, the incidence of heterogeneity in GC is much higher than that in BC (Rüschoff et al., 2012), and the heterogeneous expression of HER2 may affect the activity of trastuzumab since the nature of the non-cleavable linker does not allow bystander activity (i.e., the production of catabolic metabolites that are cytotoxic to neighboring tumor cells). Third, the authors noted that cancer patients are prone to develop resistance to T-DM1, and studies by Loganzo et al. (2015) revealed that tumor cells develop resistance with different mechanisms after long-term administration of T-DM1. In terms of the DM1 fraction, GC itself was susceptible to DM1 resistance, and several studies have shown that microtubule inhibitors (such as vinorelbine and taxane) are much less active in GC patients than in other cancers (Kulke et al., 2006; Galletti et al., 2020). Since DM1 binds to tubulin in cells, altered or mutated tubulin may also affect the response to T-DM1 (Kavallaris, 2010). Another major mechanism is the multidrug resistance (MDR) mechanism; MDR1 (also known as P-glycoprotein) is an ATP-dependent transporter that mediates the efflux of drugs and toxins in cells. This mechanism allows the drug to escape toxic effects by directly pumping the drug out of the cell before it reaches its target (Kavallaris et al., 2008). Considering T-DM1 as a whole, there are multiple mechanisms for the resistance of tumor cells to T-DM1, including inefficient internalization of the HER2-T-DM1 complex, defective endosomal trafficking after internalization, and further insufficient lysosomal degradation (Barok et al., 2014).

### 4.2 Non-small cell lung cancer

More than 80% of lung cancer (LC) patients are smokers, making LC a high-incidence disease with a high mortality rate. It principally consists of small cell lung cancer (SCLC) and non-small cell lung cancer (NSCLC) (Molina et al., 2008). NSCLC patients account for a large proportion, i.e., 85% of LC patients. Lung adenocarcinoma (LUAD) and lung squamous cell carcinoma (LUSC) are two common subtypes of NSCLC (Molina et al., 2008; Herbst et al., 2018). As a basic measure of cancer treatment, chemotherapy was proven to be effective, but due to its narrow therapeutic index and non-specific cytotoxic effect, clinical research on targeted therapy for NSCLC had commenced (Wang et al., 2022). Human epidermal growth factor-tyrosine kinase inhibitors (EGFR-TKIs), such as gefitinib, erlotinib, and afatinib, can be used in NSCLC patients with activating EGFR receptor mutations (Akamatsu et al., 2019). Various studies have shown that trastuzumab, alone or in combination with other chemotherapeutic agents, including cisplatin, gemcitabine, docetaxel, and paclitaxel, was effective in treating patients with HER2-mutated NSCLC, and it significantly improved the survival (Zinner et al., 2002; Mazieres et al., 2022). However, a majority of patients develop resistance to trastuzumab and EGFR-TKIs, and new drugs are urgently needed.

Following the discovery of HER2 receptor amplification in BC and GC, the amplification, overexpression, and mutation of the HER2 receptor have also been identified in NSCLC, and recent literature confirmed that its frequency was 2%–5%, 10%–15%, and 1%–4%, respectively (Huang et al., 2020). However, unlike BC and GC, many years ago, there were different methods for testing HER2 receptors in LC, and all of them had different criteria. The boundaries of HER2 positivity, amplification, and overexpression in LC were unclear (Bontoux et al., 2022). In most clinical practices, next-generation sequencing (NGS) was used to test HER2 gene amplification, but there is no unified standard to define the results of NGS; the gold standard for HER2 gene amplification remains ISH (Ren et al., 2022) as well as IHC staining for HER2 overexpression (defined as IHC0 or 1^+^ is HER2 negative, and IHC2^+^ or IHC3^+^ is HER2 positive). For HER2 mutational activation in LC, pathways used to estimate HER2 mutations include Sanger sequencing, NGS, and reverse-transcription polymerase chain reaction (RT-PCR) (Imyanitov et al., 2021; Ren et al., 2022). In NSCLC, HER2 mutational activation occurs in approximately 5% of patients with NSCLC, including exon 20 insertions and point mutations, both in the kinase domain (Stephens et al., 2004). HER2 mutations occur predominantly in exon 20 insertions (approximately 95%), expressed as insertions or deletions, with the *Y772dupYVMA* mutation being the most common HER2 mutation (Stephens et al., 2004; Riudavets et al., 2021).

In 2014, Daniele Cretella et al. conducted an *in vivo* and *in vitro* study to explore the role of T-DM1 in HER2-overexpressing NSCLC cell lines. They first obtained the expression of HER2 protein in individual cells by immunoblotting cell lysates from a panel of NSCLC cell lines (including H3255, Calu-6, and Calu-3). Three of these NSCLC cells, Calu-3 (very high expression of HER2 protein), H3255 (strong expression of HER2), and Calu-6 (weak expression of HER2), were selected for the study. After T-DM1 was applied to these three cell lines, significant results were obtained. T-DM1 showed a stronger antiproliferation effect in HER2-overexpressing Calu-3 cells. No obvious effect was seen after T-DM1 treatment of Calu-6 cells with low levels of HER2 expression, and intermediate results were observed in H3255 cells. Simultaneously, Daniele Cretella et al. also studied the H1781 cell line carrying the mutant HER2 (*G776insV_G/C*) and showed that the sensitivity of H1781 to drugs was not affected by the mutant receptor. Daniele Cretella mentioned that T-DM1 sensitivity is closely related to cell surface HER2 expression and ruled out the hypothesis that the differential effect of T-DM1 could be attributed to differential sensitivity to the microtubule polymerization inhibitor DM1. In subsequent experiments, it was found that T-DM1 retained natural killer cell (NK)-mediated ADCC, T-DM1 also affected the cell cycle and caused an increase in the proportion of cells in G2-M phase and a decrease in G1 and S phases, and no change in cell cycle distribution was observed in trastuzumab-treated cells. The potent targeted therapeutic effect of T-DM1 was studied by Daniele Cretella et al. They also studied the effect of T-DM1 on a gefitinib-resistant HER2-overexpressing PC9 cell line. Overexpression of HER2 tremendously heightens the efficacy of T-DM1. The inhibition of cell viability was 40% at 1 μg/mL. Decreased *AKT* and p70S6K phosphorylation was observed after 48 h of treatment with T-DM1, suggesting that T-DM1 may improve gefitinib treatment. Interestingly, Daniele Cretella et al. investigated the relationship between the *in vivo* activity of T-DM1 and tumor size by seeding mice with 4 × 106 or 8 × 106 Calu-3 cells, and the tumors were clearly visible. Trastuzumab (15 mg/kg i. p.) or T-DM1 (15 mg/kg i. v.) was administered every 6 days. The results showed that both T-DM1 and trastuzumab significantly inhibited tumor growth in small tumors. However, in large-volume tumors, trastuzumab does not express satisfactory antitumor effects compared with the control group given solvent, and there was no significant difference between the two groups. Conversely, T-DM1 showed an unexpected therapeutic effect in bulky tumors, significantly reducing tumor growth compared with the control group. In response to this result, Daniele Cretella *et al.* demonstrated that tumor cells that make up small tumors exhibit a higher degree and intensity of HER2 immunolabeling than large tumors. The experiment by Daniele Cretella *et al.* suggests that even in the case of resistance to EGFR-TKIs, T-DM1 may provide a new therapeutic strategy for NSCLC with HER2 overexpression (Cretella et al., 2014). These experimental results show that T-DM1 may be an efficacious and novel drug for treating LC with HER2 gene mutation or amplification and has a more significant therapeutic effect on cancers with HER2 mutation.

In 2017, La Monica et al. (2017) found that T-DM1 synergizes third-generation EGFR-TKI in combination treatment of EGFR-mutation-activated NSCLC cell lines. Silvia La Monica et al. treated PC9 cell lines (characterized by *E746-A750* deletion) and PC9-T790 M cell lines (characterized by *E746-A750* deletion and *T790M* mutation) (produced by exposing PC9 cells to high concentrations of gefitinib) carrying in-frame deletions in exon 19 of the *EGFR* gene with osimertinib and observed that the drug showed strong antitumor effects on those two cancer cell lines. The IC_50_ values of PC9 and PC9-T790 M cells were 14.4 ± 2.3 and 7.6 ± 0.5 nM, respectively. Flow cytometry analysis showed that the plasma membrane EGFR level was not significantly upregulated after osimertinib treatment. Conversely, in PC9 and PC9-T790 M cells, osimertinib enhanced HER2 expression on cell membranes, and this effect was positively correlated with dose and time. This result is undoubtedly suggestive of a possible interaction between osimertinib and T-DM1. Based on this result, Silvia La Monica et al. investigated the effects of the combination of osimertinib and T-DM1 on cell growth, death, and antibody-dependent cell-mediated cytotoxicity (ADCC). PC9 cells and PC9-T790 M cells were treated with 2.5 μg/mL or 1.5 μg/ml T-DM1 after 72 h of pretreatment of osimertinib, and MTT results observed additive inhibition of cell proliferation in the combination treatment group, which would reduce cancer cell proliferation only within a single utilization. In addition, the presence of IL-2-activated NK cells in PC9-T790 M cells pretreated with osimertinib significantly enhanced T-DM1-dependent cytotoxicity, implying that osimertinib may improve the efficacy of T-DM1 *in vivo*. Some studies have suggested that HER2 amplification is a mechanism of resistance to osimertinib in cancer patients (Planchard et al., 2015). This concept was also confirmed in another study by Silvia La Monica et al. An assay was performed by treating PC9 and PC9/HER2c1 cells (obtained by stable transfection of PC9 cells with a HER-2 expression vector) with high concentrations of osimertinib. The rate of proliferation of both cell types was then assessed 72 h later. HER2 overexpression was found to reduce sensitivity to osimertinib significantly. Dose–response curves of osimertinib in the presence of fixed concentrations of T-DM1 showed synergistic effects. Western blot analysis showed that combining the two drugs was much more effective than the use of a single drug. These included significant inhibition of HER2 phosphorylation and enhanced activation of AKT and MAPK pathways. To further demonstrate the significant antitumor effect of T-DM1 combined with osimertinib, Silvia La Monica et al. conducted *in vivo* experiments in which PC9/HER2c1 cells were inoculated subcutaneously into the back of BALB/c nude mice. Animals were randomly divided into four groups, and each group received different drugs (saline, osimertinib, T-DM1, or a combination of the two drugs). Osimertinib was administered orally at a dose of 10 mg/kg, and T-DM1 was administered intraperitoneally at a dose of 15 mg/kg. The results completely intersected with our expectations, and T-DM1 significantly inhibited tumor growth. Moreover, the tumor growth was completely suppressed in the two-drug combination group. The remarkable therapeutic effect of T-DM1 combined with osimertinib in *EGFR-*mutated or HER2-overexpressing NSCLC cell lines was shown by Silvia La Monica et al. and validated by several other researchers over the years. Clinical trials were conducted by Katsuyuki Hotta et al. and Bob T Li et al.

In a phase II study designed by Hotta et al. (2018), the effect of T-DM1 on HER2-positive NSCLC was investigated. Eligible patients had an IHC score of 3^+^, IHC2^+^ and FISH^+^ (median ratio of HER2 to chromosome 17 copy number = 2), and an exon 20 insertion mutation. A total of 15 patients with LC were enrolled. The trial showed that 1 of 15 patients responded to T-DM1, and the ORR was 6.7% (90% CI: 0.2%–32.0% and 95% CI: 0.3%–27.9%). No tumor reduction was observed in the subgroup of tumors with IHC score 3^+^ or IHC2^+^/FISH^+^, the median PFS time was 2.0 months (95% CI: 1.4–4.0), and the median OS time was 10.9 months. All adverse effects that occurred during the course of the experiment were known, mainly thrombocytopenia (40%) and hepatotoxicity (20%). T-DM1 did not exhibit a significant therapeutic effect in patients with HER2-positive NSCLC, contrary to the conclusions drawn from cellular and animal studies and in contrast to gastric cancer, which showed similar results. The therapeutic effect of T-DM1 in humans is significantly different from the effect of the drug on cells. The specific reason is unknown, but Katsuyuki Hotta *et al.* pointed out that this may be due to the heterogeneity of tumors. Similar to gastric cancer, NSCLC also shows a high incidence of heterogeneity, and another study pointed out that about 30% of NSCLC cases show heterogeneity (Kobyakov et al., 2015). The other reason for this effect may be the “selective” effect of T-DM1, not simply the targeting of HER2 by T-DM1, but the selective killing of strongly staining cells throughout the tumor by T-DM1, and the survival and proliferation of weakly staining tumor cells that cannot be selected by T-DM1. This study is too small and has many other limitations to answer all our questions. After this research, many clinical trials were conducted to prove the potential of T-DM1. However, the trials could not achieve satisfactory results, implying that the research of T-DM1 in NSCLC has reached a bottleneck.

However, things transformed a bit after the trial by Li et al. (2018). In this single-center, T-DM1 phase II basket trial, patients with IV LC or LC with recurrent HER2 gene mutations detected by NGS were included. Every 21 days, patients received T-DM1 at a dose of 3.6 mg/kg intravenously until disease progression or unacceptable toxicity. A total of 18 patients were screened, and the ORR was 44% (95% CI, 22%–69%) with three of 18 patients (17%) showing disease progression as the best response. Median PFS was 5 months (95% CI, 3–9 months) for all patients; meanwhile, it was 6 months (95% CI, 4 months to not reached) for responders, with the longest PFS observed (11+ months) in patients with stable disease, where the best response was 27% tumor shrinkage. The results of this trial, with stable disease in 39% of the patients, include disease control lasting more than 11 months, which has not been observed in the previous clinical trial, demonstrating the efficacy of T-DM1 and providing insight into the future of drug development in this field. Previous studies have used IHC staining as a criterion based on experience in breast cancer and gastric cancer. For T-DM1 to work, tumor cells need to exhibit HER2 overexpression, making sense to apply this rule to LC. However, this study reveals that HER2 IHC may not be a desirable biomarker for LC. Numerous studies have shown that mutations activate the EGFR, and this discovery led to the approval of many cancer gene-targeted therapies, which altered cancer treatment worldwide. HER2*-*activating mutations have shown promising therapeutic targets, and next-generation sequencing should be more widely used in evaluating NSCLC.

Based on the experimental results of Bob t li et al., Solange Peters conducted a multicenter, single-group trial (trial registration NCT02289833) to investigate the efficacy and safety of T-DM1 in patients previously treated for HER2-overexpressing metastatic NSCLC (Peters et al., 2019). Patients with HER2-positive (IHC2^+^ or 3^+^), and with HER2 mutations detected by NGS, were eligible for the trial. The study enrolled 49 patients and divided them into two cohorts, IHC2^+^ (n = 29) and IHC3^+^ (n = 20). Cancer patients received T-DM1 at a dose of 3.6 mg/kg intravenously every 3 weeks, until investigator-assessed disease progression, uncontrolled toxicity, or study termination. The results showed that in the IHC 2^+^ cohort, there was no treatment response; eight out of 29 patients (28%) had stable disease (SD), 16 (55%) had disease progression (PD), and five out of 29 patients could not be evaluated (17%); in the IHC3+ cohort, the ORR was 20% (95% CI, 5.7%–43.7%); four out of 20 patients (20%) achieved partial response (PR), four patients (20%) achieved SD, 11 patients (55%) achieved PD, and one patient (5%) died before the first planned tumor evaluation. In IHC2^+^ and 3^+^ cohorts, the median PFS was 2.6 months (95% CI, 1.4–2.8) and 2.7 months (95% CI, 1.4–8.3), respectively. The median OS was 12.2 months (95% CI, 3.8–23.3) and 15.3 months (95% CI, 4.1-Not Estimable), respectively. These results indicate that patients with IHC3+ tumors had objective responses, whereas no responses were observed in the IHC2+ cohort. Interpretation of these results is limited due to a small number of patients enrolled and fewer patients in each molecular subgroup. Therefore, the NCT02675829 basket trial results further validate and support the potential antitumor effects of T-DM1 in advanced NSCLC characterized by HER2 oncogenic alterations.

In a recent study, Iwama et al. (2022) explored the role of T-DM1 in patients with NSCLC whose HER2 was activated by an *ex vivo* exon 20 insertion mutation, which is detected by NGS. Patients with histologically or cytologically confirmed stage III or IV LUAD or positive HER2 exon 20 insertion mutation tests were included in the study. The 21 patients enrolled received intravenous infusions of T-DM1 (3.6 mg/kg) every 21 days. Of these 21 patients, 11 (52.4%) had a reduction in tumor size from baseline, of whom eight (38.1%) had a partial response and three (14.3%) had SD. Thus, the ORR and disease control rates were 38.1% (90% CI, 23.0%–55.9%, *p* = 0.2091) and 52.4% (90% CI, 35.2%–69.0%), respectively. The median PFS was 2.8 months (95% CI, 1.4–4.4 months), and the median OS was 8.1 months (95% CI, 3.5–13.2 months); a significant response was observed in this study consistent with that observed in Bob T li’s study (ORR 38.1%). In addition, the data showed that the incidence of adverse events of grade 3 or higher was low throughout the trial, and T-DM1 is expected to be a potential therapeutic drug for such mutations. Despite the excellent efficacy of T-DM1 for this mutation, nearly half of the patients still do not respond to the drug, so new biomarkers are desirable to improve the efficacy of NSCLC with this mutation.

### 4.3 Colorectal cancer

Colorectal cancer (CRC) is a serious disease that is common in both men and women. According to the statistics, there were nearly 1.9 million new cases in 2020, and 930,000 people died of colorectal cancer in the same year, accounting for about one in 10 cancer cases and deaths (Sung et al., 2021). After years of efforts, although improved screening, solvability, and awareness of CRC increased the number of nonmetastatic cases, combinations of molecular therapies (including bevacizumab, cetuximab, panitumumab, aflibercept, and regorafenib) and other cytotoxic agents have doubled the chances of surviving CRC. However, as for metastatic colorectal cancer (mCRC), approximately a quarter of CRC patients were diagnosed at stage IV; thus, identifying new therapies and therapeutic advances in the field became crucial (Afrăsânie et al., 2019). In mCRC, approximately 6% of patients reported HER2 gene amplification and protein overexpression, but only 4% of patients showed activated HER2-receptor tyrosine kinase mutations (Seo et al., 2014; Parikh et al., 2017). Moreover, HER2 overexpression was also detected in CRC in different studies, varying from 0.5% to 40% (Essapen et al., 2004; Kruszewski et al., 2010; Ingold Heppner et al., 2014). A recent study proves that HER2 may also be an ideal target in CRC, leading to unexpected effects. This study points to the need for further preclinical studies and clinical trials, and consistently, favorable treatment response was observed in patients with HER2-targeted amplification cancer in clinical trials; this study increases the feasibility of the HER2-targeted CRC treatment strategy (Mangiapane et al., 2022). Multiple case reports have observed tumor shrinkage and clinical benefits in colorectal cancer patients who were treated with T-DM1, suggesting its potential in CRC (Haslem et al., 2017; Parikh et al., 2017; Sandhu et al., 2020). In 2020, Chung et al. (2020) conducted a cellular experiment to evaluate the effects of T-DM1 on colon cancer and its relationship with metformin in improving the efficacy of T-DM1 through caveolin-1-mediated endocytosis. In this study, three colon cancer cell lines (SW48, HT-29, and LS174T) were used. Among them, LS174T highly expressed HER-2; HT-29 and LS174T were BRAF- and KRAS-mutated, respectively; and SW48 was wild-type colon cancer cells with high expression of EGFR. Cetuximab, trastuzumab, and T-DM1 were added to the three types of colon cancer cells, respectively. The effects of cetuximab, trastuzumab, and T-DM1 on the growth and apoptosis of the cancer cells were detected after 72 h of incubation. The results showed that cetuximab had a significant influence on SW48 cells, but HT-29 and LS174T cells were resistant to it. In addition, treatment of colon cancer cells with trastuzumab plus T-DM1 showed a synergistic effect, with a stronger inhibitory effect than that of T-DM1 alone, especially LS174T cells, which showed high HER2 expression and had better sensitivity. Moreover, the number of apoptotic cells also increased in the LS174T group, which was not observed in all three groups at the same cell level even when treated with high trastuzumab concentration (10 μg/mL). To further verify the effects of T-DM1 on HER2*-*positive colon cancer cells, Yuan-Chiang Chung et al. performed an *in vivo* experiment in which SW48, HT-29, and LS174T cells were subcutaneously transplanted, constructing a xenograft nude mouse model. The results showed that cetuximab inhibited the growth of SW48 xenograft tumors and had no effect on HT-29 and LS174T tumors. In contrast, trastuzumab combined with T-DM1 showed considerable efficacy and could effectively inhibit the growth of HT-29 and LS174T tumors. In addition, the levels of anti-apoptotic and survival markers were also significantly reduced in the tumors. The immunohistochemical (IHC) studies of tissue sections also showed a decrease in the proliferation marker Ki67 in the combination therapy group. This study indicated that T-DM1 did have a therapeutic potential in HER2-positive colon cancer, but clinical trials were still needed for further confirmation.

Furthermore, the HERACLES-B trial was designed based on the success of the previous HERACLES trial (Sartore-Bianchi et al., 2016). The HERACLES trial is a proof-of-concept, multicenter, open-label, phase 2 trial that was performed at four academic cancer centers in Italy to evaluate the efficacy of trastuzumab and lapatinib in patients with HER2-positive, KRAS exon 2 (codons 12 and 13) wild-type mCRC. Of the 27 enrolled patients, 59% (95% CI 39–78) achieved disease control, and no serious adverse effects were observed during the course of the trial. It demonstrated that trastuzumab plus lapatinib had been shown to be efficacious in patients with HER2*-*positive mCRC, none of whom are sensitive to chemotherapy and anti-EGFR antibody therapy. Moreover, it highlighted the importance of trastuzumab as a potential molecular targeted therapy in colorectal cancer.

HERACLES-B is a multicenter, open-label, phase 2 trial involving patients with histologically confirmed RAS wild-type mCRC (KRAS exons 2, 3, and 4; *NRAS* gene exons 2, 3, and 4 positive). Moreover, HER2+ CRC patients were included in this study and tested positive for IHC and FISH (Sartore-Bianchi et al., 2020). The primary objective of this study was to investigate the role of pertuzumab plus T-DM1 in HER2^*+*^mCRC, which was administered in cycles, with pertuzumab administered on day 1 of cycle 1 and T-DM1 on day 1 of every three cycles. A total of 31 patients were included. The results showed that three patients achieved partial response, and the ORR was 9.7% (95% CI: 0–28). A total of 21 patients (67.7%, 95% CI: 50–85) achieved SD, and the disease control rate was 77.4%. The median PFS was 4.1 months (95% CI: 3.6–5.9). By applying statistics, researchers found that in the cohort of patients with HER2 IHC score 3^+^, the PFS was 5.7 months, but in the cohort of patients with HER2 IHC score 2^+^, the PFS was only 1.9 months (HR 0.20, 95% CI: 0.07–0.56, *p* = 0.0008). This study concludes that patients with higher HER2 IHC scores have longer PFS. This association was also observed for objective response rates and long-term stable disease (*p* = 0.03). Although T-DM1 can show a certain antitumor effect, the effect is inadequate. There is still a long way to go for T-DM1 to show excellent efficacy in CRC, such as through specific compatibility or chemical modification, which needs to be improved by scholars.

### 4.4 Ovarian and uterine cancer

Ovarian cancer (OC) is an extremely severe disease among women. According to the statistics, 240,000 women are diagnosed with OC each year, with the 5-year survival rate lower than 45%, causing 150,000 deaths (Webb and Jordan, 2017). The histological types of OC mainly include the serous type, mucinous type, endometrioid type, and clear cell type. HER2 overexpression was also observed in ovarian cancer, with a HER2-positive rate of 3.8% by IHC and 5.7% by SISH (Chung et al., 2019). The American Society for Clinical Oncology and the College of American Pathologists states that HER2 IHC testing should be combined with FISH testing in OC patients. This approach effectively reduces the false-negative rate of HER2 status in mucinous ovarian cancer, and the corresponding HER2-positive detection rate is effectively increased (Hodeib et al., 2015). The efficacy of molecular targeted therapy for ovarian cancer was investigated as early as 2013, but the results with trastuzumab were unsatisfactory. In the GOG-160 trial, 41 patients with epithelial OC underwent trastuzumab treatment at a dose of 4 mg/kg weekly, administered over 90 min, and showed an ORR of only 7.3% (Bookman et al., 2003). Yu et al. (2014) conducted an *in vivo* and *in vitro* experiment to explore the role of T-DM1 in HER2-positive human OC cells, using OC cells SKOV3, ES-2, A2780s, A2780cp, PA-1, and HO8910 and BC cell lines SKBR-3 and MCF-7. Repeated experiments exhibited high expression of HER2 protein in SKOV3 and SKBR-3 cells, low expression in other cells, and almost no HER2 protein expression in ES-2 OC cell lines. After T-DM1 treatment, the results showed that T-DM1 had a strong suppressive effect on HER2-overexpressing SKOV3 and SKBR-3 cells, but had a weak inhibitory effect on HER2-negative cells (ES-2 and MCF-7). IC_50_ values exceeded 1,200 nmol/L and 720 nmol/L, respectively. The results of the *in vivo* study showed that the inhibitory effect of T-DM1 on the xenograft model was concentration-dependent. The low dose (3 mg/kg) of T-DM1 showed no inhibitory effect on tumor growth, while the moderate dose (10 mg/kg) of T-DM1 presented complete tumor remission in three out of five mice without regrowth. Tumor regression was complete in mice treated with a high dose (30 mg/kg) of T-DM1. This phenomenon persisted for such a long time that no tumor regrowth was observed throughout the treatment. T-DM1 demonstrated impressive antitumor activity in this study, directing later targeting studies in ovarian cancer. Nicoletti et al. (2015) stated that T-DM1 showed surprising efficacy against ovarian and uterine carcinosarcoma (CS). Nicoletti et al. (2015) established a total of eight uterine and ovarian CS cell lines (SARARK-1, 3, 4, 5, 6, 7, 8, and 9) from fresh tumor biopsy samples. HER2 was highly expressed in SARARK-6 (IHC3^+^ and FISH^+^) and low expressed in SARARK-1 (IHC0/1^+^ and FISH^−^). SARARK-6 cells were plated in six wells and treated with T-DM1 and trastuzumab 24 h later and then counted after 72 h by flow cytometry. The results showed that, unlike trastuzumab, T-DM1 showed a more potent antiproliferation effect on SARARK-6 cells. They found a mean number of viable cells ±SEM of 17.98% ± 2.73% versus 81.75% ± 1.31% for T-DM1 versus trastuzumab (*p* < 0.0001). Moreover, this function was positively correlated with the HER2 expression in the cells. Furthermore, Roberta Nicoletti et al. established a CS xenograft tumor model by injecting SARARK-6 cells subcutaneously into mice that received five intraperitoneal injections of T-DM1 (15 mg/kg/week), trastuzumab (15 mg/kg/week), and vehicle. T-DM1 showed a potent antitumor effect *in vivo*, and the tumors were clearly visible in all mice before the drug treatment. After treatment with T-DM1, the tumors of the mice achieved complete remission compared with the other two groups (*p* = 0.0001 and *p* < 0.0001, respectively). In fact, during the study period (more than 90 days), mice in the T-DM1 group had no detectable tumors after treatment with T-DM1. In addition, Menderes et al. (2017) evaluated the antitumor activity of T-DM1 versus trastuzumab and pertuzumab in epithelial ovarian cancer (EOC). They established 10 primary EOC cell lines. The results showed that the cytotoxicity of T-DM1 was significantly more powerful than that of trastuzumab and pertuzumab *in vitro*. The mean percentage of viable cells +SD for trastuzumab alone was 68.54 ± 7.82, that of pertuzumab alone was 49.80 ± 4.51, that of trastuzumab combined with pertuzumab was 33.24 ± 3.34, and that of T-DM1 was 3.01 ± 0.38. *In vivo* results showed that compared with mice treated with trastuzumab alone (*p* = 0.02), pertuzumab alone (*p* = 0.03), and trastuzumab combined with pertuzumab (*p* = 0.004), T-DM1-treated mice showed a significant survival benefit.

Uterine cancer (UC) is the sixth most common cancer in women and the 14th most common cancer worldwide. Uterine serous carcinoma (USC) usually occurs in postmenopausal women and is a rare histological type of endometrial cancer (accounting for approximately 10%). As an aggressive malignancy, it is characterized by poor prognosis with few treatment options, and the treatment effect of both surgery and radiotherapy/chemotherapy is unsatisfactory (Artioli et al., 2015). HER2 overexpression is also seen in USCs. Several studies have shown that 6% of USCs express HER2 IHC3^+^ and 15% of USCs express FISH^+^ (Jenkins et al., 2022). A preclinical study has revealed that T-DM1 seems to have a therapeutic effect on primary HER2-overexpressing USCs. In 15 USC cell lines established from fresh tumor biopsy samples, 2.5 μg/mL of T-DM1 and trastuzumab induced potent ADCC in all HER2*-*overexpressing USC primary cells (mean cytotoxicity ±SEM, 61.6% ± 5.3% vs. 58.4% ± 5.74% (T-DM1 vs. trastuzumab). Interestingly, T-DM1 was more potent than trastuzumab in inhibiting the proliferation of USC cells. T-DM1 achieved the same experimental results as observed by Roberta Nicoletti et al.; all 490 animals in the T-DM1 group had no detectable tumors after T-DM1 treatment (English et al., 2014). In 2016, Alessandro D Santin et al. reported a clinical case of T-DM1 in a patient with HER2-overexpressing metastatic USC, who was resistant to both radiotherapy and chemotherapy, and found clinical activity of T-DM1 in USC patients (Santin et al., 2017). However, the current research on T-DM1 and USC is limited to preclinical studies, and it remains unclear whether T-DM1 is effective for USC. Although the preclinical trials of T-DM1 have achieved promising results, these results still need to be verified in some well-designed large trials before they can be widely accepted.

### 4.5 Bladder cancer

Bladder cancer (BCa) is a common urinary tract malignancy. According to statistics, there were more than 57,000 new BCa cases in 2020 and more than 20,000 deaths from BCa in the same year (Sung et al., 2021). In all BCa patients, HER2 overexpression was present in 9.2% of patients, and HER2 amplification was present in 5.1% of patients (Laé et al., 2010). Studies of molecular targeted therapy for BCa began 10 years ago, and clinical phase 2 studies showed that patients with HER2-positive advanced urothelial carcinoma are sensitive to trastuzumab (Hussain et al., 2007). However, so far, the research on T-DM1 remains in preclinical studies. Tetsutaro Hayashi et al. used IHC and FISH to screen BCa cells RT112, UM-UC14, and RT4V6 with high HER2 expression, among which RT4V6 had the highest HER2 expression. The antitumor effect of T-DM1 was different from that of trastuzumab in RT4V6 cells. At the concentrations of 1 μg/mL for both T-DM1 and trastuzumab, the growth inhibition effect of T-DM1 was higher than that of trastuzumab. Moreover, T-DM1 can also increase the number of subG0/G1 and G2/M cells, and this implies that G2/M cell cycle arrest induces apoptosis. These results indicate that T-DM1 is superior to trastuzumab in inhibiting HER2*-*overexpressing BCa cells. Interestingly, this study also found that cisplatin-resistant bladder cancer cells exhibit higher HER2 expression and higher T-DM1 sensitivity, and overexpression of HER2 may contribute to development of metastatic tumors (Hayashi et al., 2015). This is the same result obtained in another study, in which HER2 amplification was significantly higher in patients with lymph node metastasis of urothelial BCa than in patients with primary tumors (Fleischmann et al., 2011). The immense achievement of T-DM1 in the preclinical study of bladder cancer suggests that T-DM1 is more efficacious than trastuzumab in the targeted therapy of bladder cancer, and these studies have laid the foundation for the research of T-DM1 in bladder cancer. However, the role of T-DM1 in bladder cancer has not been clinically validated, and discrepancies between preclinical and clinical studies are also frequent in other cancer fields. To define the efficacy of T-DM1, several well-planned clinical studies are essential.

### 4.6 Biliary tract cancer

Biliary tract cancer (BTC) is an aggressive and heterogeneous disease. Anatomically, BTCs are categorized into intrahepatic cholangiocarcinoma (ICC), extrahepatic cholangiocarcinoma (ECC), distal cholangiocarcinoma (dCCA), gallbladder carcinoma (GBC), and ampullary carcinoma (Tella et al., 2020). For patients with advanced and unresectable BTCs, systemic chemotherapy, such as gemcitabine plus cisplatin, is the standard of care (Valle et al., 2017). However, the therapeutic effect of chemotherapy is unsatisfactory. Targeted therapy for BTCs has been started recently. HER2 gene alterations were present in 5.4% of patients with BTCs, including 2.7% with HER2 amplification and 2.3% with HER2 mutation (Mondaca et al., 2019). Studies have shown that the rate of HER2 overexpression in ECC (17.4%) changes from that in ICC (4.8%) (Galdy et al., 2017). Milind Javle et al. conducted the MyPathway clinical trial. MyPathway is a multicenter, open-label, phase 2a, multiple basket study conducted in 2021. This study explored the antitumor activity of pertuzumab in combination with trastuzumab and its safety in patients with metastatic HER2-positive BTC. The average response rate with trastuzumab-targeted therapy was superior to that with second-line chemotherapy, and even though the sample size of MyPathway is too small to conclude that trastuzumab and pertuzumab have therapeutic activity in BTC, this trial provides hope for BTC targeted therapy (Javle et al., 2021). The role of T-DM1 in BTC was first investigated in 2019, and Yoriko Yamashita-Kashima et al. conducted a preclinical study of T-DM1 in BTC cells; six cholangiocarcinoma cell lines (KKU-100, KMCH-1 *etc*.); seven gallbladder carcinoma cell lines (Mz-ChA-1, OCUG-1, *etc*); and four ampullary carcinoma cell lines (TGBC-18-TKB, TGBC-50-TKB, TGBC-51-TKB, and TGBC-52-TKB) were used in the study. The sensitivity of KMCH-1 to T-DM1 was about one-tenth of that of BC cell lines after treatment with T-DM1. Nevertheless, the growth inhibitory activity in BTC cells was correlated with the level of HER2 expressed by the cells (R2 = 0.6127). Subsequently, Yoriko Yamashita-Kashima et al. established KMCH-1, Mz-ChA-1, OCUG-1, and KKU-100 BTC cell line xenograft tumor models in nude mice. The results showed that HER2 status in KMCH-1, Mz-ChA-1, OCUG-1, and KKU-100 models was HER2-positive, HER2-positive, HER2-negative, and HER2-negative, respectively. T-DM1 was administered every 3 weeks, KMCH-1 and Mz-ChA-1 models in all three medium-dose groups, and the OCUG-1 model observed significant antitumor effects in the high-dose group of T-DM1, but not in the KKU-100 model. Yoriko Yamashita-Kashima et al. showed that T-DM1 acts in BTC cells in a similar way as in BC cells, including cell cycle arrest and inducing apoptosis (Yamashita-Kashima et al., 2019). Trastuzumab combined with pertuzumab has achieved a surprising therapeutic effect in BTC. However, the role of T-DM1 cannot be ignored in the targeted therapy of BTC. The experimental results of Yoriko et al. may lead to new treatment options for BTC patients. However, these results still need to be verified in clinical trials. Recently, scholars reported a case of a HER2-amplified ECC patient who achieved partial response to T-DM1 targeted HER2 therapy, and the patient achieved partial response after 1 week of T-DM1 treatment (Mondaca et al., 2019). Although one clinical case report cannot be considered as proof that T-DM1 is effective, the results are hoped to encourage scholars to pursue further research in this direction.

## 5 Conclusion and perspective

In the clinical trial, the basic cancer treatment regimen is traditional radiotherapy and chemotherapy (Mun et al., 2018), but with serious drug resistance emerging as a major challenge for cancer treatment, it gradually cannot meet the clinical needs (Nussinov et al., 2021). As a first-line drug for HER2+ BC, T-DM1 has significantly improved the survival of HER2*+* BC patients (Ferraro et al., 2021). Recent studies confirmed that T-DM1 had a significant antitumor effect on other HER2*+* cancers, both *in vitro* and *in vivo*, and the clinical trials were also consistent, showing great therapeutic value (Cretella et al., 2014; Thuss-Patience et al., 2017). Despite this, we have to point out that T-DM1 had a poor therapeutic effect on the HER2*+* NSCLC patients, even though its effect was observed in clinical patients with HER2-mutated NSCLC (Hotta et al., 2018). Some reasons did exist affecting the clinical trial results of T-DM1 on HER2+ GC (Ishimine et al., 2015; Peng et al., 2015). T-DM1 still showed the same therapeutic effect as taxanes on HER2*-*positive GC, and Mark Barok and Yoriko Yamashita-Kashima et al. have shown the therapeutic potential of T-DM1 on HER2-positive GC cells *in vitro and in vivo* (Barok et al., 2011; Yamashita-Kashima et al., 2013). Peter C Thuss-Patience et al. attributed the difference in results to discordant HER2 expression between the primary tumor and the metastatic tumor or after first-line chemotherapy for metastatic disease with or without trastuzumab, the molecular profile of the tumor being altered, and HER2 may be altered and lost during progression. Another study by Manish A Shah et al. partially explains this theory. The loss (or low expression) of the HER2 receptor on gastric cancer cells does affect the action of T-DM1, but as a result, the therapeutic effect of T-DM1 on gastric cancer patients with low HER2 expression is even less effective than that of taxanes. Only in patients with HER2-positive gastric cancer, T-DM1 expression results in the same efficacy as that of taxanes. The author believes that this discrepancy in clinical studies is most likely due to the second reason proposed by Peter C Thuss-Patience et al. Because of the high incidence of heterogeneity in gastric cancer, the heterogeneous expression of HER2 may affect the action of T-DM1. Moreover, this heterogeneous expression of HER2 may not be detected by conventional methods, such as IHC staining or ISH. Therefore, the clinical study of T-DM1 in patients with HER2-positive GC seems to have entered a bottleneck period, and the identification of the heterogeneous expression of GC and the changes in HER2 may become the future research direction in this concern. In addition to T-DM1, several other novel ADCs have been developed, including DS-8201. DS-8201 achieved a significantly higher objective response rate than chemotherapy (51% vs. 14%, *p* < 0.001) in patients with HER2 expression, and locally advanced or metastatic gastric or gastroesophageal junction cancer (Shitara et al., 2020). The stronger inhibitory effect of DS-8201 on HER2 heterogeneous expression in GC than on T-DM1 may be related to the higher cytotoxic payload concentration of DS-8201 in target cells (DAR approximately equal to 8) than T-DM1 (DAR3.5) (Xu et al., 2019). Studies have shown that one of the resistance mechanisms of T-DM1 is related to the low cytotoxic payload concentration in the target cells (Vernieri et al., 2019), which sheds light on and provides ideas for the future development of T-DM1, implying that improving the cytotoxic payload concentration of T-DM1 in target cells may be a promising direction. For ovarian cancer, uterine cancer, bladder cancer, and biliary tract cancer, T-DM1 has been shown to have potent antitumor effects, observed in preclinical studies. However, preclinical studies are insufficient, and more clinical trials should be designed to prove the role of T-DM1 in treating corresponding cancers. The clinical utilization of T-DM1 needs further exploration before its application on other types of HER2+ cancers, but we should not neglect its great therapeutic potential, as was affirmed by various studies, as better than trastuzumab in cancer-targeted therapy and did well for breast cancer treatment.
